# From Data to Cure: A Comprehensive Exploration of Multi-omics Data Analysis for Targeted Therapies

**DOI:** 10.1007/s12033-024-01133-6

**Published:** 2024-04-02

**Authors:** Arnab Mukherjee, Suzanna Abraham, Akshita Singh, S. Balaji, K. S. Mukunthan

**Affiliations:** Department of Biotechnology, Manipal Institute of Technology, Manipal Academy of Higher Education, Manipal, India

**Keywords:** Multi-omics, Big data, Machine learning, Targeted therapeutics, Network pharmacology

## Abstract

In the dynamic landscape of targeted therapeutics, drug discovery has pivoted towards understanding underlying disease mechanisms, placing a strong emphasis on molecular perturbations and target identification. This paradigm shift, crucial for drug discovery, is underpinned by big data, a transformative force in the current era. Omics data, characterized by its heterogeneity and enormity, has ushered biological and biomedical research into the big data domain. Acknowledging the significance of integrating diverse omics data strata, known as multi-omics studies, researchers delve into the intricate interrelationships among various omics layers. This review navigates the expansive omics landscape, showcasing tailored assays for each molecular layer through genomes to metabolomes. The sheer volume of data generated necessitates sophisticated informatics techniques, with machine-learning (ML) algorithms emerging as robust tools. These datasets not only refine disease classification but also enhance diagnostics and foster the development of targeted therapeutic strategies. Through the integration of high-throughput data, the review focuses on targeting and modeling multiple disease-regulated networks, validating interactions with multiple targets, and enhancing therapeutic potential using network pharmacology approaches. Ultimately, this exploration aims to illuminate the transformative impact of multi-omics in the big data era, shaping the future of biological research.

## Introduction

In the era of targeted therapeutics, drug discovery approaches emphasize the underlying disease mechanisms, accompanied by target identification and lead discovery. This targeted therapeutic system focuses on molecular perturbations, has become critical in the drug discovery process [1], and is entirely reliant on big data to transform the current understanding and accessible data into valuable information employed to enhance clinical outcomes [2]. Biological and biomedical research and its applications have infiltrated a big data era due to the heterogeneity and enormity of omics data [3]. There is a growing acknowledgment among researchers regarding the significance of adeptly integrating various strata of omics data, referred to as multi-omic studies. The modeling and exploration of the intricate interrelationships among diverse omic layers have the potential to unveil crucial functional and clinical insights. The transformative omics revolution observed in biological research after the inception of genomic sequencing has engendered an extensive corpus of data, concurrently fostering technologies that facilitate the cost-effective and streamlined quantification of biological molecules on a large scale. Presently, omics technologies have undergone substantial expansion to encompass more unbounded methodologies, such as assays reliant on next-generation sequencing (NGS) and mass spectrometry. Tailored assays now exist for each stratum of molecular activity, spanning genomes to metabolomes. Researchers investigate the field of genomics through techniques like whole-genome or whole-exome sequencing, explore transcriptomics using RNA-seq [4], delve into epigenomics via methodologies such as bisulfite sequencing (BS-seq) [5], ChIP-seq for histone modifications [6], and ATAC-Seq for open chromatin [7]. The three-dimensional conformation of the genome can be elucidated through techniques like Hi-C or chromatin interaction analysis with paired-end tag (ChIA-PET) [8, 9].

Additionally, researchers may investigate proteomics and delve into metabolomics, predominantly employing mass spectrometry [10]. These methodologies have transformed biomedical investigations by furnishing a more thorough understanding of the studied biological system and the molecular intricacies inherent in the progression of diseases. The prodigious volume of data generated in biomedicine necessitates the employment of sophisticated informatics techniques to glean novel insights, advance our understanding of diseases, enhance diagnostic capabilities, and formulate individualized therapeutic strategies [11]. Within this framework, machine-learning (ML) algorithms have emerged as among the most auspicious methods in the field. Omics data have the potential to refine classification beyond a simplistic dichotomy of healthy versus diseased, offering substantial clinical benefits. These methodologies can enhance patient treatment by aligning with the distinctive biology of their specific ailment, categorizing patients into subtypes, or positioning them along a spectrum of disease manifestation. These datasets also contribute significantly to an enhanced comprehension of the pathogenic mechanisms underlying diseases and biomarkers. We briefly reviewed how the accessibility of these omics data have transformed biology and aided in developing systems biology to comprehend the biological phenomena [12] and provide a platform for integrating these high-throughput data, which focuses on targeting and modeling multiple disease-regulated networks to screen leads and validating their interactions with multiple targets for enhanced therapeutic potential [13].

This review aims to explore the transformative impact of omics data in the big data era of biological and biomedical research. Focusing on multi-omic studies, it seeks to model and understand the intricate interrelationships among diverse omic layers to unveil functional and clinical insights. The expansion of omics technologies has provided tailored assays for each molecular stratum, revolutionizing biomedical investigations. Leveraging ML algorithms, the study aims to refine disease classification, enhance diagnostics, and develop personalized therapeutic strategies, ultimately contributing to an improved understanding of pathogenic mechanisms and biomarkers.

## Multi-omics Data

### Genomics

Genomic investigations have constituted a principal methodology in elucidating the etiology of diseases and delineating potential targets for treating various complex diseases. The statistical analyses conducted in these studies frequently encounter challenges related to multiplicity, faint signals, and the inherent interdependence among genetic markers [14]. ML algorithms can be implemented to address these challenges. These algorithms uncover subtle patterns and relationships by improving the sensitivity and specificity of the analysis that may not be evident through conventional statistical approaches. Yu et al. illustrated the effectiveness of an integrative co-localization (INCO) algorithm designed for the seamless integration of single-nucleotide variants (SNVs) and copy number variations (CNVs). This algorithm facilitated the synthesis of the genetic variations, yielding a more precise and refined genetic region. The refined region enhanced the accuracy in identifying causal variants associated with the studied biological phenomena [15]. This allows the identification of specific genetic mutations or alterations associated with diseases. Also, Liu et al. demonstrated a comprehensive analysis of these genetic variations, which resulted in the identification of disease-causing variants in 10 of the 16 investigated rare diseases. Notably, the analysis revealed new potentially pathogenic variants for two disorders. For the first time, clinical whole genome sequencing (WGS) successfully identified a causative simple sequence repeat (SSR) variation associated with Machado–Joseph disease, highlighting the power of clinical WGS in providing molecular-level diagnostic clarity for rare diseases [16]. By unraveling the intricate genomic landscape, targeted therapies can be precisely tailored to address the underlying genetic anomalies, paving the way for personalized treatment strategies that consider individual genetic variations.

### Transcriptomics

Transcriptomics pertains to the assessment of the entire repertoire of genes expressed as transcripts or mRNAs and non-coding RNAs [17]. Alterations in the gene expression profile often occur under diseased conditions. Transcriptomic methodologies, such as microarrays and high-throughput sequencing, facilitate the systematic monitoring of the entire transcriptome [18]. This comprehensive approach allows for the acquisition of a global cellular signature or fingerprint, offering insights into the dynamic changes in gene expression associated with various biological states [19]. Moreover, it establishes a critical bridge between genomics and proteomics by elucidating the intricate connection between the transcriptome and the subsequent protein expression patterns [20]. In targeted therapies, understanding transcriptomic changes is crucial for identifying key genes and pathways involved in disease progression. By employing these techniques, researchers unveiled the dysregulated genes and designed interventions that modulate gene expression levels, ultimately contributing to the development of more effective and tailored therapeutic approaches [21–23].

### Epigenomics

The dysregulation of epigenetic processes is a pivotal factor in the onset and advancement of human diseases. Due to the dynamic nature of the intricately regulated epigenetic marks and mechanisms, these modifications can serve as discernible biomarkers [24]. Differential DNA methylation can lead to a spectrum of disorders, encompassing inflammatory conditions, precancerous lesions, and malignancies [25–27]. Moreover, Kikutake and Yahara, in 2016, conducted a genome-wide study that illustrated the advantage of ChIP-Seq and RNA-Seq over microarrays in encompassing histone modification regions not addressed by microarrays. The study delved into the exploration of associations between histone modifications and the occurrence of aberrant gene expression during the progression of the disease [28]. Comprehending epigenetic modifications is crucial for identifying reversible alterations that influence gene expression in targeted therapeutics. A more sophisticated approach to therapy can be achieved by developing medicines that selectively affect the activity of genes linked to disease progression by focusing on these epigenetic alterations [29].

### Proteomics

Aberrant regulation of protein function plays a critical role in disease pathogenesis, highlighting the imperative goal of biomedical research to comprehend the perturbation of the proteome in disease progression. While transcriptome data, specifically mRNA abundance, cannot infer protein abundance accurately, direct assessments of protein function become essential [30]. Although conventional methodologies often concentrate on individual proteins or a limited set, recent progress in sample separation and mass spectrometry technologies allows for the holistic consideration of a complex biological system as an integrated unit [31]. The swift progress in proteomics experimental techniques has spurred the development of diverse downstream bioinformatics analyses. These analyses contribute significantly to elucidating the intricate relationships between molecular-level protein regulatory mechanisms and phenotypic manifestations, particularly in the context of disease initiation and progression [32]. Ohlsson et al. analyzed differential protein expression, utilizing statistical methods to identify analytes specific to autoimmune diseases that perturb immunoregulatory responses [33]. By identifying dysregulated proteins, researchers can design therapies that specifically target these molecular players, addressing key components of the disease mechanisms. This nuanced understanding of the proteomic landscape enhances the precision and efficacy of targeted therapeutic interventions.

### Metabolomics

Metabolic profiles offer a detailed understanding of physiological states and are highly susceptible to genetic and environmental perturbations. Fluctuations in metabolic profiles can offer insights into the mechanisms underlying pathological conditions, thereby serving as potential biomarkers for the diagnosis and evaluation of the risk associated with disease onset [34]. Large-scale metabolomics data sources are abundant as high-throughput technologies continue to evolve. For instance, meticulous statistical and bioinformatic analysis of intricate metabolomics data are crucial for achieving accurate and significant findings that are applied in real-world clinical settings [35, 36]. Metabolites serve as biomarkers and contribute to an enhanced comprehension of the pathophysiology underlying various diseases [37–40]. In targeted therapies, metabolomics helps identify disease-associated metabolic signatures, shedding light on altered biochemical pathways [38, 41]. This knowledge guides the development of therapeutic agents that target-specific metabolic processes, addressing the unique metabolic needs of diseased cells and contributing to more efficient and accurate therapeutic strategies.

## Exploitation of Omics Data

**Fig. 1:**
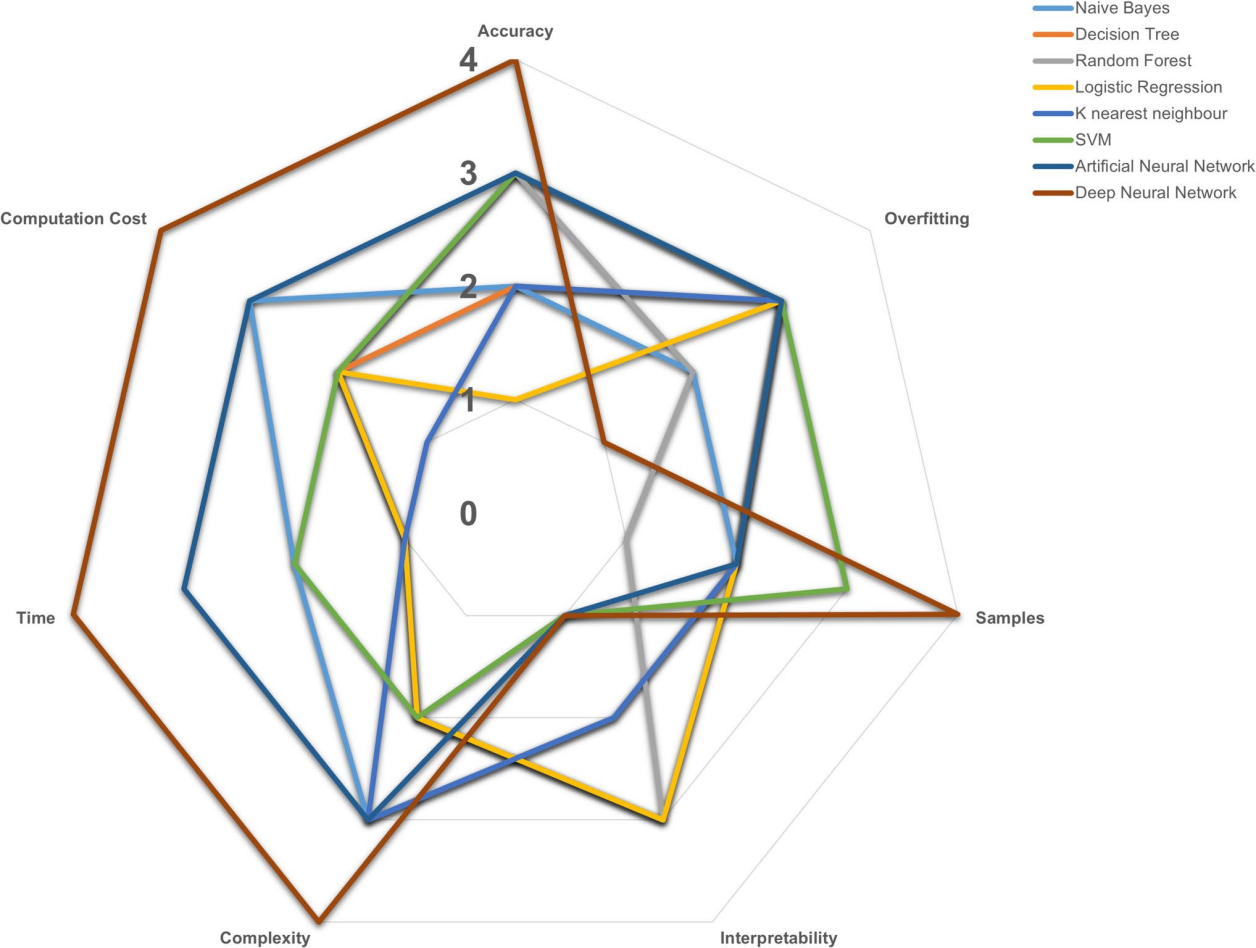
Overview of prevalent ML algorithms with attribute rankings [1—Low, 2—Medium, 3—High, 4—Very High] for concise representation

**Fig. 2:**
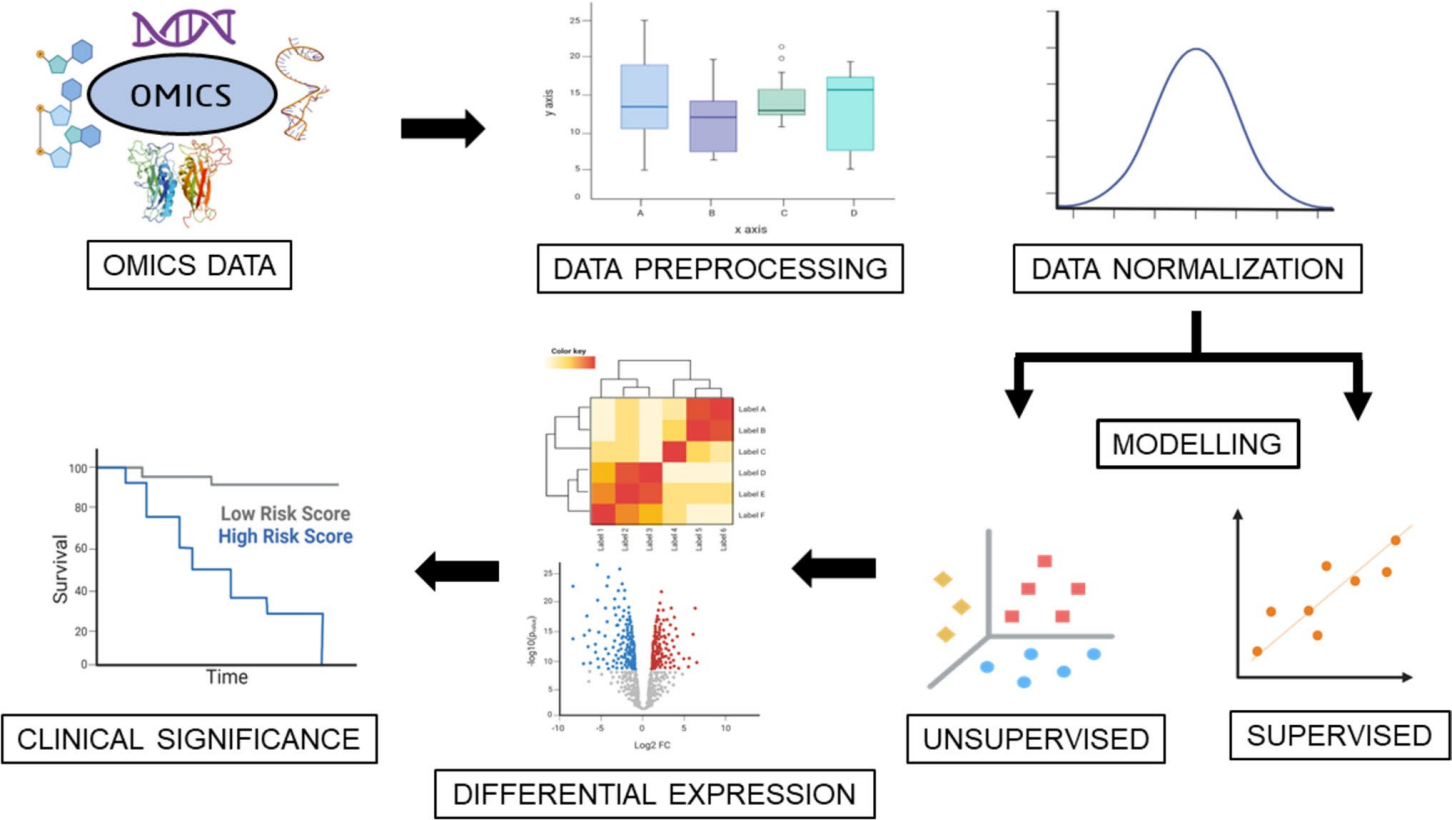
Steps associated with implementing different strategies for integrating and analyzing multi-omics data for cancer biomarker discovery

The high-dimensional structure of omics data raises several barriers to acquiring information. Data processing, normalization, integration, and analysis of these high-dimensional data have gained attention among researchers through several computational techniques such as ML, meta-heuristic, and statistical approaches [42]. The exponential increase in the volume of data generated through high-throughput sequencing and related methodologies necessitates the application of statistical models capable of extracting precise and interpretable predictions from the wealth of biological information [11]. ML models exhibit enhanced performance when trained on large datasets, making them well-suited for the intricate integration of multi-omics data in bioinformatics applications [43]. The speed, accuracy, interpretability, computing cost, complexity, and sample sizes were pragmatically scored in the range of 1–4, with low to very high as a threshold for supervised ML algorithms by Reel et al. [44], as demonstrated in Fig. 1. These ML models have accelerated the drug development process by identifying novel biomarkers, detecting early prognostic biomarkers, predicting their clinical significance, identifying mutational patterns, and determining gene expression cohorts. The steps in implementing the different techniques in integrating and analyzing multi-omics data for cancer biomarker discovery are illustrated in Fig. 2 [45]. Applying these different algorithms for feature selection and classification and integrating high-dimensional heterogeneous omics data provides potential inference methods in disease progressions, which are listed in Table 1.

**Table 1:** The use of multiple ML approaches on OMICS datasets to identify novel biomarkers, early prognostic biomarkers, and prediction of clinical significance

Omics data type	Assessment	Machine learning models	References
Transcriptomic	• Prognostic biomarkers identification • Recurrence risk • Intrinsic and extrinsic features of the disease environment • Expression cohorts	• Autoencoder and Network fusion • Monte-Carlo feature selection (MCFS) • Support Vector Machine (SVM) • Principal component and similarity measurements	[22, 102, 105–107]
Metabolomic	• Metabolic biomarkers in understanding disease pathophysiology • Targeted therapeutic targets • Pharmacogenomic responses	• Principle component analysis and K-means clustering • SVM • Random Forest • KNN • Naïve Bayes for classifier models	[78, 108, 109]
Genomics	• Risk prediction • Genetic mutation • Mutational distribution • Cancer somatic variants • Copy number variation • Understanding the genetic and molecular mechanisms	• Random Forest • ANN • Logistic regression • SVM • CNN • DNN	[110–113]
Proteomics	• Survival risk prediction • Protein levels, modifications, and interactions • Biomarkers	• Linear models • PCA • K-Means clustering	[114, 115]
Epigenomics	• DNA methylation patterns • Histone modifications • Therapeutic response • Alternative splicing prediction • Disease classification	• PCA • Naïve Bayes • SVM • Random forest • RNN	[90, 116–118]

### Supervised

Supervised ML algorithms are employed to assess labeled datasets. These supervised methods analyze input and output data across diverse classifications to train models. These models, once trained, are subsequently utilized to make predictions on multi-omics datasets. For instance, they can be applied to identify the data characteristics that underpin-specific biomarkers [46]. Supervised learning algorithms are well-suited for addressing two fundamental problems, which include classification and regression [42]. In the context of classification problems, the output variable is distinctly discrete, necessitating the categorization of diverse groups or categories of biomarkers with distinct molecular features that are significantly altered between healthy and diseased states [47]. In contrast, regression problems involve output variables characterized by continuous real values, such as estimating the survival risk in disease progression [48]. The versatility of supervised learning algorithms positions them as valuable tools capable of effectively handling both discrete categorization and continuous outcome prediction tasks.

#### Logistic Regression

Logistic regression (LR) is a robust supervised classification method, particularly applied in omics data analysis to identify and prioritize relevant biomarkers dynamically, optimizing the model’s predictive accuracy and interpretability in the context of targeted therapies. Functioning as an extension of ordinary regression, LR models dichotomous variables, estimating the probability of an instance belonging to a specific class ranging from 0 to 1 [49]. They are extensively employed in assessing the associations between biomarkers and binary outcomes and can be extended to handle the integration of diverse omics data types. Also, the LR algorithm can seamlessly incorporate these diverse data types by treating them as covariates in the model, allowing the integration of multiple datasets. However, omics data frequently exhibits high interdependence among features, violating the assumption of independence in LR [50], leading to unstable coefficient estimates and reduced interpretability. Researchers face the decision of categorizing biomarkers as either continuous or categorical covariates. Predicting the survival risk of the biomarkers with skewed distributions necessitates normalization for integration as continuous covariates, often accomplished through log transformation. Many biomarkers with skewed distributions align well with the Normal curve on the log scale, recommending log transformation for obtaining dependable odds ratio (OR) estimates predicting clinical events [51]. Biomarkers identified through the omics datasets encounter challenges in reproducibility owing to the inherent heterogeneity stemming from diverse platforms or laboratory settings [52]. These inconsistencies served as a catalyst for the development of resilient biomarker identification methodologies through the integration of multiple datasets. The distinct constant terms in LR gauged the sample heterogeneity. Through the minimization of variations in constant terms within a given dataset, this approach effectively preserved both intra-dataset homogeneity and inter-dataset heterogeneity [53].

#### Support Vector Machines

Support vector machine (SVM) stands as a prevalently used and robust ML algorithm in the field of bioinformatics, particularly employed for classification and regression tasks. The principle of SVM revolves around identifying a hyperplane, either a line or a plane in high-dimensional space that maximizes the separation between distinct classes with the maximum margin. The crucial data points nearest to this hyperplane, termed support vectors, wield significant influence over its positioning. Researchers employ SVM on Omics data for biomarker discovery, leveraging its sensitivity and flexibility. The application involves metabolite ranking through a systematic reduction of biological replicates to assess the influence of sample size on biomarker reproducibility and robustness. This methodology reflects SVM’s effectiveness in handling Omics data intricacies, emphasizing its utility in the dynamic field of biomarker identification and characterization [54]. SVM exhibits adaptability to both linear and non-linear data, proving especially efficacious when the number of features far surpasses the number of samples. Despite its versatility, SVM presents a spectrum of advantages and limitations, with its performance intricately linked to the judicious choice of kernel and the dataset’s size [55]. In multi-omics datasets, which often involve intricate interactions [56], SVMs present an advantageous method for detecting non-linear patterns that might elude linear techniques like logistic regression, utilizing kernel functions. These kernel functions facilitate the transformation of input data into higher-dimensional feature spaces, where the previously non-linear associations between molecular attributes and clinical outcomes can potentially become linearly separable. This transformation enables SVMs to construct complex decision boundaries that may not be achievable within the original input space. Commonly utilized kernel functions such as the radial basis function (RBF) kernel and polynomial kernel empower SVMs to capture the intricate interactions among molecular features [57]. Moreover, Ensemble methods, such as bagging and boosting, can be integrated with SVMs to improve predictive performance and robustness [58]. Consequently, SVMs offer a robust avenue for uncovering concealed patterns and relationships within multi-omics datasets, thus fostering advancements in targeted therapy strategies.

#### Decision Tree

Decision tree (DT) stands as one of the earliest and most prevalent ML algorithms, representing decision logic in a tree-like structure to classify data. The tree comprises nodes with multiple levels, starting with the root node at the top. Internal nodes, conducting tests on input variables, guide the classification process toward child nodes based on test outcomes. This iterative process continues until it reaches a leaf node, which signifies the decision outcome. Decision trees are valued for their interpretability, quick learning, and frequent integration into medical diagnostic protocols. During sample classification traversal, the outcomes of tests at each node offer sufficient information to infer its class [59]. The DT undergoes initial training on “ground truth” data, learning to establish decision boundaries for the most crucial features based on class grouping. In the context of biomarkers, classes may represent cases and controls, with protein levels serving as the features. Established methodologies exist to identify the features contributing most significantly to class differentiation. This not only enhances interpretability but also mitigates the risk of classifying proteins that lack medical relevance, avoiding potential confounding factors related to distinct sample handling in cohort groups [60]. DT can handle the non-linear relationships within omics data analysis by employing recursive partitioning. This process entails dividing the data into subsets according to predictor variable values. At each node of the tree, decision trees discern the predictor variable and its corresponding split point that can optimally segregate the data into homogeneous groups relative to the outcome variable [61, 62]. Through iterative data splitting based on predictor variables, decision trees construct a hierarchical framework adept at modeling non-linear interactions among molecular features in omics datasets [63]. This adaptability renders decision trees well-suited for delving into complex relationships and identifying significant biomarkers or predictors within intricate biological systems.

#### Random Forest

The random forest (RF) algorithm stands out as a premier method for classification in ML due to its accuracy in handling substantial datasets. It also operates as a learning algorithm constructing multiple decision trees during training. The collective predictions of individual trees contribute to the model’s overall output. RF operates as a tree predictor, with each tree relying on random vector values, showcasing its efficacy in enhancing the robustness and predictive capabilities crucial for accurate classification in bioinformatics applications [64]. In omics analysis, employing systematic, data-driven feature selection methods is crucial to avoid biased selection. RF, in conjunction with other methods, has proven effective in tasks such as gene and metabolite selection for disease prognosis and progression [65–68]. RF inherently captures non-linear relationships between molecular features and clinical outcomes, making it well-suited for omics data analysis through partitioning the feature space into decision regions. RF can identify complex patterns and interactions that may not be apparent with linear models, mitigating the risk of overfitting and noise often encountered in high-dimensional omics datasets [69, 70]. This can be achieved by employing bagging, an ensemble method, to enhance predictive accuracy and mitigate overfitting. This technique entails generating multiple bootstrap samples from the original dataset and training multiple decision trees on each sample. Through the integration of predictions from numerous trees, RF diminishes variance and achieves enhanced generalization to new data, thereby averting overfitting [71]. Additionally, boosting, a sequential ensemble method, is employed, with subsequent models learning from preceding misclassified models. Algorithms like AdaBoost and Gradient Boosting Machines iteratively refine the model, assigning greater importance to misclassified instances, leading to improved overall performance and diminished overfitting [71, 72]. The integration of these ensemble methods with random forest can yield more resilient models that exhibit superior generalization to high-dimensional omics data while minimizing the susceptibility to overfitting associated with specific training data patterns. Existing studies often focus on specific omic types, lacking stable feature selection procedures for power calculations in identifying biomarkers. However, the lack of assessment and validation of the identified markers hinders their utility in study design or power analysis for translational research.

#### Naïve Bayes

The naïve bayes (NB) classifier, a probabilistic learning model rooted in the Bayes theorem, is used for classification tasks. This algorithm relies on the assumption of feature independence, making predictions about an instance’s class by calculating the class prior probability and the likelihood of that specific class. The NB classifier’s foundation in probability theory renders it valuable for diverse classification bioinformatics applications [73]. The algorithm can be described using: $P\left(X|Y\right)=P\left(Y|X\right) P(X)/P(Y)$. The *P*(*X*|*Y*) represents the Posterior, indicating the probability of *X* being true given that *Y* is true. On the other hand, *P*(*Y*|*X*) describes the likelihood of a class, representing the probability of *B* being true given that *A* is true. Additionally, *P*(*X*) and *P*(*Y*) denote the prior probability of a class and a predictor, respectively. NB algorithms find application in the selection of genetic biomarkers through the concurrent examination of genome-wide SNP data and large omics data [74, 75]. NB algorithm has not been extensively explored for predicting biological classes. However, refined Bayesian classification methods that consider dependencies among features have demonstrated precise predictions of biological classes. NB effectively handles noisy and irrelevant features in the high-dimensional data by leveraging its probabilistic framework. Noisy features have minimal impact on conditional probabilities, as NB considers joint probability distributions. Similarly, irrelevant features insignificantly affect class probability estimation, as NB prioritizes discriminating between classes based on informative features in the omics data [76]. Nevertheless, enhancing the robustness of predictions is achieved by employing an ensemble approach, specifically bagging, with Naïve Bayes classifiers. This strategy enhances the effectiveness of ranking and selecting attributes used by each bagged classifier, ultimately reinforcing attribute independence in the biomarker selection process [77].

#### K-Nearest Neighbours

K-nearest neighbors (KNN) is a versatile and efficient ML algorithm suitable for classification and regression tasks. It classifies an unknown sample based on its proximity to the *K*-nearest samples in the training set, assigning the most common class among these neighbors. As a lazy learning algorithm, KNN stores training data during the training process, conducting actual classification or regression at the prediction stage for enhanced speed and memory efficiency. Despite its simplicity, adaptability to linear and non-linear data, and ease of implementation, KNN’s performance depends on parameters like the selection of *K*, feature scale, and the relevance of features [55]. The KNN algorithm is applied to discern patterns within high-dimensional omics datasets, classify or predict sample phenotypes based on proximity in feature space, and aid in identifying potential biomarkers by revealing similarities among biological samples. Researchers exploit KNN’s adaptability and simplicity to explore intricate omics relationships, contributing to biomolecular marker discovery in precision medicine [78–80]. KNN’s non-parametric nature allows it to capture complex relationships and patterns within multi-omics datasets without making strong assumptions about data distribution [81]. This attribute is particularly valuable in deciphering intricate molecular landscapes associated with disease phenotypes, areas where conventional linear models may encounter limitations. KNN is frequently employed for imputing missing metabolite abundances in omics datasets [82]. Nevertheless, it is crucial to acknowledge that KNN operates under the assumption that missing values are uniformly distributed at random across the dataset, a premise that does not align with the typical characteristics of metabolomics data [83]. Despite this consideration, the algorithm’s versatility in handling classification and regression tasks integrated with Artificial Intelligence (AI) methodologies can enhance its utility for uncovering novel insights in personalized healthcare [84].

#### Artificial Neural Network

Artificial neural networks (ANNs) draw inspiration from biological neural networks, resembling interconnected artificial neurons. These artificial neurons receive input, undergo a data transformation, and produce an output, mirroring the functional principles of their biological counterparts. The ANN model does not make any assumptions about the distribution of data before the learning process, enhancing the versatility and applicability of ANNs across various domains [85]. ANNs comprise input and output layers connected by hidden layers. Input nodes transmit information to hidden nodes through activation functions, while hidden nodes activate based on presented evidence. Weighting functions in hidden layers process evidence, and when node values reach a threshold, outputs are generated. ANNs require extensive training data, limiting application in rare events with insufficient data. They do not accommodate human expertise substitution for quantitative evidence. The key advantages of employing ANNs are that they exhibit robust fault and failure tolerance, scalability, and reliable generalization capacity, enabling accurate prediction or classification of novel, ambiguous, and unlearned data, which makes it suitable for biomarker studies, contributing to the development of biomarker panels that, when used collectively, enhance prognostic capabilities in diseases. ANNs trained on large-scale multi-omics datasets can be adapted and transferred to new domains or disease contexts with limited labeled data. Transfer learning techniques enable the transfer of knowledge learned from one dataset to another, accelerating model training and improving the predictive performance of ANNs in multi-omics data analysis [86]. However, ANNs can be integrated with dimensionality reduction methods like autoencoders or t-distributed stochastic neighbor embedding (t-SNE) to reduce the dimensions of omics data while retaining critical features. This enables the visualization of high-dimensional data in lower-dimensional spaces, which assists in deciphering intricate molecular relationships [87].

#### Deep Neural Networks

Deep neural networks (DNNs) serve as potent tools for data-driven modeling in bioinformatics. Comprising layers with interconnected nodes and edges that encapsulate mathematical relationships, DNNs undergo iterative refinement via backpropagation during training. Post-training, these updated relationships function as predictive equations, enabling the accurate forecasting of output variables based on input variables. An inherent strength of DNNs lies in their ability to capture and express intricate relationships within a system, irrespective of its non-linear and complex nature [88]. The intricate and interlinked hierarchical representations of training data utilized by deep neural networks for estimating purposes render the comprehension of these estimates exceptionally challenging, resulting in low explainability. However, we identified two deep learning neural networks, Convolution Neural Networks (CNNs) and Recurrent Neural Networks (RNNs) that are widely used for Omics integration and analysis. The CNNs are found to recognize spatial patterns in sequences and are efficient for identifying relevant genetic markers [89]. Moreover, RNNs capture temporal dependencies in time-series omics data, aiding in discerning dynamic biomarker patterns [90]. The significance of DNNs in omics data analysis is underscored by the burgeoning advancements in multi-omics technologies and the accumulation of extensive bio-datasets with issues like overfitting, interpretability deficits, data heterogeneity integration challenges, and the need for enhanced prediction accuracy. Integrated approaches with dimensionality reduction methods are established to extract targets from each omics data and construct sample similarity networks based on feature matrices. Subsequently, the fused similarity networks undergo training in a DNN, significantly reducing data dimensionality and mitigating the risk of overfitting [91]. This advancement holds promise for the evolution of targeted therapy.

### Unsupervised

Unsupervised ML is employed for the data lacking labels. The model uncovers latent patterns by identifying groups of samples sharing common characteristics. The speed and reliability of the unsupervised ML methods depend on the nature of the data and are sensitive to outliers. Method selection balances computational efficiency and captures specific patterns in complex data. However, Accuracy is gauged by the likelihood of the model generating a dataset under a specified distribution. Unsupervised learning aims to cluster data, reveal natural groupings, or establish associations among data points within large databases [92]. Unsupervised multi-omics methods primarily focus on classifying diseases and sample subtypes while identifying biomarkers or modules associated with a disease. Current methods often handle multiple outcome variables individually instead of using multivariate models.

#### Principle Component Analysis

Dimension reduction is vital for downstream tasks like pattern recognition, classification, and clustering in high-dimensional data [55]. While various techniques exist, principal component analysis (PCA) stands out as a classical and widely employed method. PCA offers optimal linear projection in Euclidean space with eigenvectors representing weighted linear combinations of features [93]. PCA can function as a valuable asset for quality control and the mitigation of batch effects in multi-omics investigations. Through the visualization of data distributions and the detection of outliers or batch effects, PCA empowers researchers to evaluate data quality and ensure consistency across various experimental batches or sample groups [94]. Consequently, this process contributes to bolstering the reliability and reproducibility of downstream analyses. Identifying principal components associated with disease phenotypes can prioritize molecular features that contribute most significantly to the observed variation, guiding the selection of potential targets. Acknowledging that only a subset of features is dominantly relevant in genomic transcriptomic studies, using all features may compromise robustness and interpretability in high-dimensional data. Therefore, Park et al. introduced an integrative analysis of multi-omics data, utilizing blockwise sparse principal components to alleviate multi-collinearity and redundancy, achieve dimensionality reduction, and identify crucial variables, unlike other multi-omics integration methods for biomarker discovery [95].

#### K-Means Clustering

The K-means clustering divides a data space into k clusters, assigning each data point to the cluster with the nearest mean value. The clustering involves partitioning data into groups with similar characteristics, particularly focusing on geometric proximity in the feature space. This approach aids in creating a learning problem for precise recovery of cluster centroids, eliminating impractical considerations [96]. K-means clustering is sensitive to the initial selection of cluster centroids, which can lead to suboptimal solutions and influence cluster assignments [97]. Additionally, it operates under the assumption of equal variance among clusters, which may not hold for multi-omics data with varying levels of noise and biological variability. To address these limitations, performing multiple initializations of K-means with different seed values and opting for the solution exhibiting the lowest within-cluster variance can mitigate the sensitivity to initialization, enhancing the robustness of clustering outcomes. Integrating semi-supervised learning techniques with K-means clustering can leverage labeled data and prior knowledge in multi-omics analysis, improving the interpretability and accuracy of subtype identification and biomarker discovery [98]. Moreover, recent research has delved into the analysis of multilayer data and similar studies, demonstrating enhanced predictive capabilities for disease outcome models compared to single-layer analyses. K-Means primarily segregate distinct data types but struggle to group various interconnected omics measurements into cohesive clusters [99, 100].

#### Autoencoders

The autoencoder functions as a neural network that aims to replicate the input signal, capturing essential features of the data and restoring its original form. It employs a hidden layer as both an encoder and decoder, ensuring consistency between the encoded and decoded data. Autoencoders often utilize greedy layer-wise pretraining for unsupervised learning. Commonly employed in scenarios with limited labeled data, autoencoders serve omics data exploitation well, given the challenges of obtaining labeled omics data, typically characterized by high dimensionality [101, 102]. Adversarial training generates adversarial examples that encourage the autoencoder models to acquire more discriminative and stable representations of multi-omics data, particularly in the presence of noise and variability [103]. However, interpreting the latent features learned by autoencoders can be challenging, as they often represent abstract combinations of molecular attributes. This lack of interpretability may hinder the biological insights gained from clustering analysis and limit the utility of autoencoders on omics datasets. Therefore, implementing regularization techniques such as dropout and weight decay can mitigate overfitting and prevent the model from learning noise and irrelevant patterns in the data, which can enhance the robustness of clustering outcomes [104].

## Multi-omics Data Visualization and Analysis

**Fig. 3:**
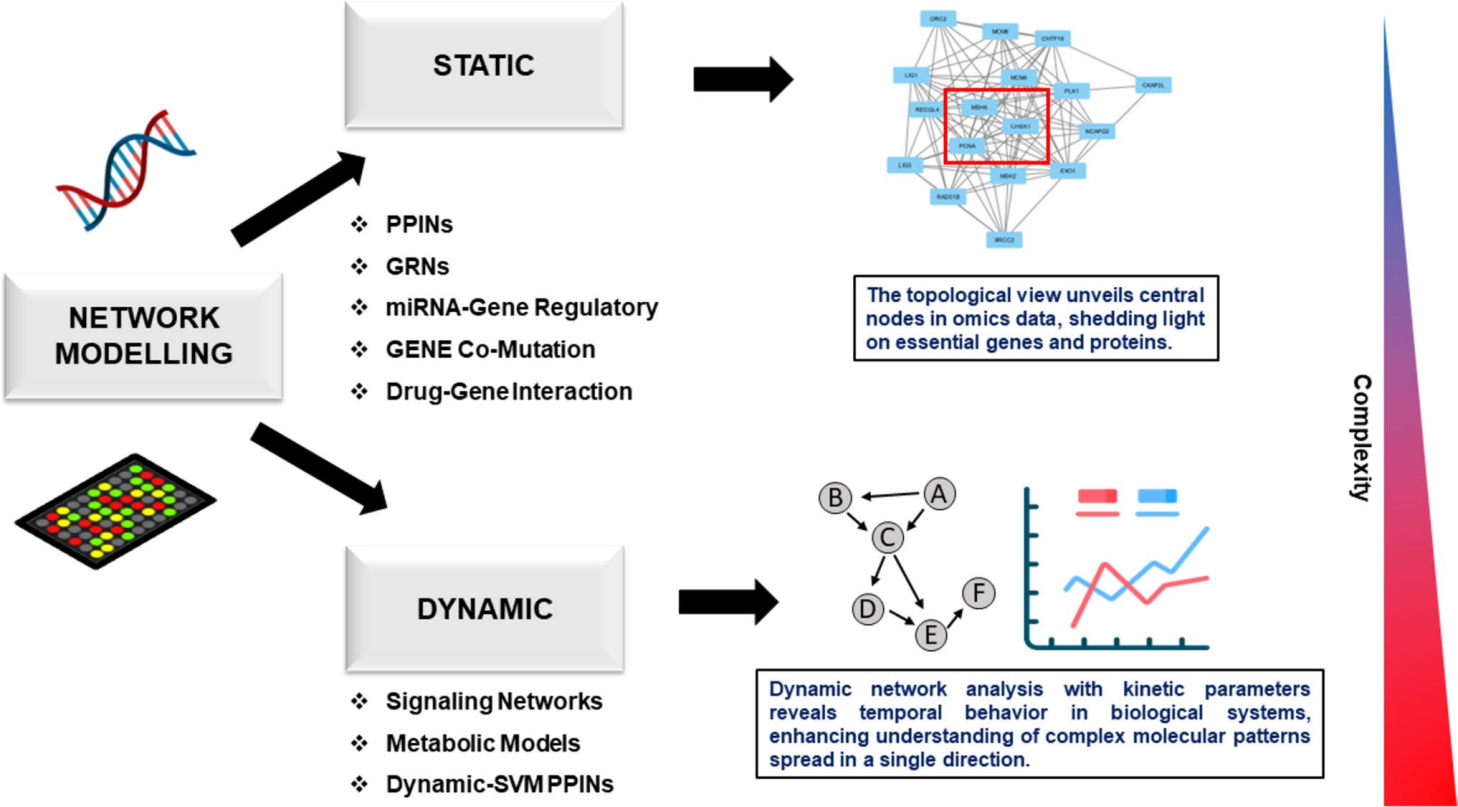
Classification of network models integrating multi-omics data and depicting the complexity of the models

Drug target identification and prioritization are critical challenges in the pre-clinical stages of pharmaceutical research. Computational techniques can reap the benefits of relatively large human genomics and proteomics data to identify targets, minimizing the time and cost. The paradigm of the “one drug target–one disease” approach in drug discovery has become inefficient. The emergence of phenotypic resilience and network topology by breakthroughs in systems biology strongly suggests that precisely selective molecules may have lower clinical efficacy than multi-target treatments. The network pharmacology approach has lately acquired prominence as a strategy for integrating omics data and developing multi-target drugs, respectively [119]. Networks can be defined at many levels of complexity (Fig. 3). Protein–protein interaction, gene regulatory, and metabolic networks are common examples of biological networks. The network concept is sometimes expanded to include drug–drug interaction in modern pharmacological research. Diverse forms of data will lead to distinct network features in terms of linkage, complexity, and structure, with edges and nodes possibly conveying many layers of data. Polypharmacology is more appropriate for complex diseases that involve complex target networks and biological pathways [120]. Network pharmacology finds diverse applications in addressing research questions. It serves various purposes, such as uncovering disease-causing candidate genes, revealing disease-associated subnetworks and systematic perturbations, and capturing therapeutic responses for efficient target identification and drug discovery. Integrating omics data into static and dynamic network models in cancer enables the identification of key molecular players, dysregulated pathways, and potential therapeutic targets. These models can also predict drug responses, guide personalized treatment strategies, and explore novel therapeutic interventions. Table 2 lists different cancer network models with their applications in integrating omics data.

**Table 2:** The applications and tools employed for different network models with integration of omics data for prognostic biomarkers and therapeutic target identification

Network model	Omics data integration	Applications	Tools	References
Protein–Protein Interaction Networks (PPINs)	Integration of gene expression, protein abundance, or proteomics data with PPINs	Identifying key proteins, functional modules, and signaling pathways associated. Discovery of potential therapeutic targets and biomarkers	• STRING (Search Tool for the Retrieval of Interacting Genes/Proteins) • Dynamic Protein–Protein Network with Support Vector Machine (DPPN-SVM) (https://github.com/brown-2/mis_localization)	[142, 143]
Gene Regulatory Networks (GRNs)	Integration of gene expression, transcription factor binding, and miRNA expression data with GRNs	Understanding gene regulation mechanisms. Identification of dysregulated pathways and regulatory modules	NetworkAnalyst (https://www.networkanalyst.ca/)	[144, 145]
Signaling Pathway Networks	Integration of phosphoproteomics, transcriptomics, or proteomics data with signaling pathway networks	Investigation of dysregulated signaling pathways. Identification of potential therapeutic targets	CNORode—a logic-based ordinary differential equation (https://github.com/saezlab/CNORode)	[135, 146]
Metabolic Networks	Integration of metabolomics, fluxomics, or transcriptomics data with metabolic networks	Understanding altered metabolic pathways. Identification of metabolic rewiring and potential targets for intervention	• metabolic Context-specificity Assessed by Deterministic Reaction Evaluation (mCADRE) (https://github.com/jaeddy/mcadre) • Reconstruction, Analysis, and Visualization of Metabolic Networks (RAVEN)	[147–150]
Co-expression Networks	Integration of gene expression or transcriptomics data with co-expression networks	Identification of functionally related genes. Discovery of co-expression patterns and potential biomarkers	• Weighted Gene Co-Expression Network Analysis (WCGNA) • CoExp	[151–154]
miRNA-gene Regulatory Networks	Integration of miRNA expression and gene expression data with regulatory networks	Investigation of miRNA-mediated regulatory interactions. Identification of dysregulated miRNAs and their target genes	NetworkAnalyst (https://www.networkanalyst.ca/)	[145, 155]
Gene Co-mutation Networks	Integration of mutation data (e.g., somatic mutation, copy number variations) with co-mutation networks	Identification of co-occurring genetic mutations and understanding the landscape of genetic alterations	Pajek (http://vlado.fmf.uni-lj.si/pub/networks/pajek/)	[156, 157]
Drug–gene Interaction Networks	Integration of drug response data, gene expression, or genomic data with drug–gene interaction networks	Prediction of drug response and identification of potential drug targets in cancer treatment	Drug–Gene Interaction Database (DGIdb) (https://www.dgidb.org/)	[158, 159]

### Static Networks

The advent of high-throughput omics technologies has provided abundant data that can be integrated into static network models to enhance our understanding of complex diseases. Omics data from genomics, such as somatic mutations and copy number alterations, can be integrated into static network models to identify driver genes and elucidate disrupted signaling pathways [121–123]. Gene expression profiles derived from transcriptomics data enable the construction of gene co-expression networks, unraveling functional modules and identifying potential biomarkers associated with specific cancer subtypes or stages [124–126]. By incorporating proteomics data, static network models can capture protein–protein interactions, shedding light on key protein hubs, signaling cascades, and potential therapeutic targets [127]. Integrating metabolomics data into metabolic network models allows for identifying altered metabolic pathways, facilitating the discovery of metabolic vulnerabilities and potential avenues for therapeutic intervention [128]. Computational approaches, including correlation-based algorithms and mutual information analysis, enable the inference of regulatory relationships and interactions between molecular components based on omics data [129]. Advanced visualization techniques, such as network graphs, aid in comprehending the intricate structure of static network models. Network analysis methods, such as centrality measures and module detection algorithms, assist in identifying critical nodes, dysregulated pathways, and functional modules within the network [130, 131]. A set of molecular regulators (genes or parts of genes) that interact with one another and other components in the cell to control the levels of gene expression of mRNA and proteins, which define the cell’s function, is called gene regulatory networks. GRNs have significant challenges, such as the delay between transcription and translation and a lack of knowledge of the necessary topology to depict the targeted phenotype and kinetics [132].

Static network models provide a comprehensive systems level view of cancer’s intricate molecular interactions and regulatory relationships, integrating multiple layers of omics data. This facilitates the generation of testable hypotheses concerning the functional roles of genes, proteins, and pathways, thereby aiding in the discovery of novel therapeutic targets and biomarkers. Static network models lack temporal information and fail to capture the dynamic changes in molecular interactions over time, impeding a comprehensive understanding of disease progression and treatment responses. They possess limitations regarding coverage of the entire molecular landscape, potentially overlooking critical genes, proteins, or interactions. They often oversimplify the heterogeneity and contextual variability, treating it as a homogeneous entity and neglecting the diversity across disease subtypes or individual patients.

### Dynamic Networks

Dynamic network models have emerged as powerful tools for understanding temporal and disease-specific dynamics. By integrating multi-dimensional omics data into these models, researchers can capture the intricate regulatory events, signaling pathways, and molecular interactions that drive disease progression and therapeutic responses. Differential equations and logic functions are generally used to generate dynamic systems [133, 134]. Dynamic models assist in evaluating the cumulative effect of dysregulated molecular mechanisms in an individual, guiding therapeutic choice in a targeted approach. Simulating metabolic and regulatory networks usually entails non-linear and dynamic models [135]. However, due to technical constraints, such models typically focus on one or a few metabolic pathways: for example, a dearth of reaction kinetics and mechanism data, the high computing complexity of non-linear parameter estimation, and a scarcity of dynamic experimental data for simulation validation [136].

On the other hand, Stoichiometric models are usually genome-wide and generated from metabolic networks, allowing them to be easily combined with high-throughput data. Genome-scale metabolic models (GEMs) are conceptual mathematical models that assist the investigators in estimating the metabolic flux rates to determine metabolic alterations to meet increased energy needs for growth, survival, rapid proliferation, and other features of tumor cells in various cancer pathogenesis through simulation studies and hypothesis testing [137, 138]. Also, cancer cells can be precisely targeted using genetically engineered therapeutics that take control of specific driving pathways through gene interaction networks (GINs) that emphasize an unbiased assessment of dysregulated pathways and uncover genetic variation that may be used to design targeted therapeutics [139].

Molecular assessment at the systems level requires capturing the static protein activity in the cell, with all interactions uncovered being integrated into a single data structure to derive protein–protein interaction networks (PPINs). Recent systems biology trends aim to develop tailored PPINs representing specific conditions [140]. Zhang et al. generated dynamic PPINs by integrating high-throughput and gene expression data to determine certain proteins’ active probability and time points. Dynamic PPINs, in contrast to static PPINs, may efficiently express both dynamic, active, and topological information in PPINs [141]. The dynamic models require time-resolved or context-specific omics data, which can be challenging to obtain and subject to noise and experimental limitations. It requires parameter estimation, such as the initial conditions, reaction, or decay rates [128]. Estimating these parameters accurately from limited experimental data can introduce uncertainty into the model. Inaccurate or uncertain parameter values can affect the model’s predictive power and limit its ability to capture the true dynamics of the biological system. Addressing these flaws and limitations requires continuous improvement in data collection techniques, computational algorithms, and integration of multiple omics data types. Efforts to incorporate more comprehensive and context-specific data, consider non-linear relationships and account for variability across diseases and patients can enhance the accuracy of the dynamic network models.

## Integrative Approach for Probing Disease Mechanisms

Biological processes and molecular functions arise from intricate interactions among thousands of molecules, constituting inherent complexity. Integration of metabolomics data with other omics data holds significant promise for achieving a holistic understanding of disease mechanisms. Metabolomics, which focuses on the comprehensive analysis of small molecule metabolites within biological systems, provides unique insights into the functional status and metabolic phenotypes associated with various physiological and pathological conditions [160, 161]. The integration of omics datasets with computational models and network analysis tools elucidates the complex interplay between genes, proteins, metabolites, and cellular processes underlying disease phenotypes.

**Fig. 4:**
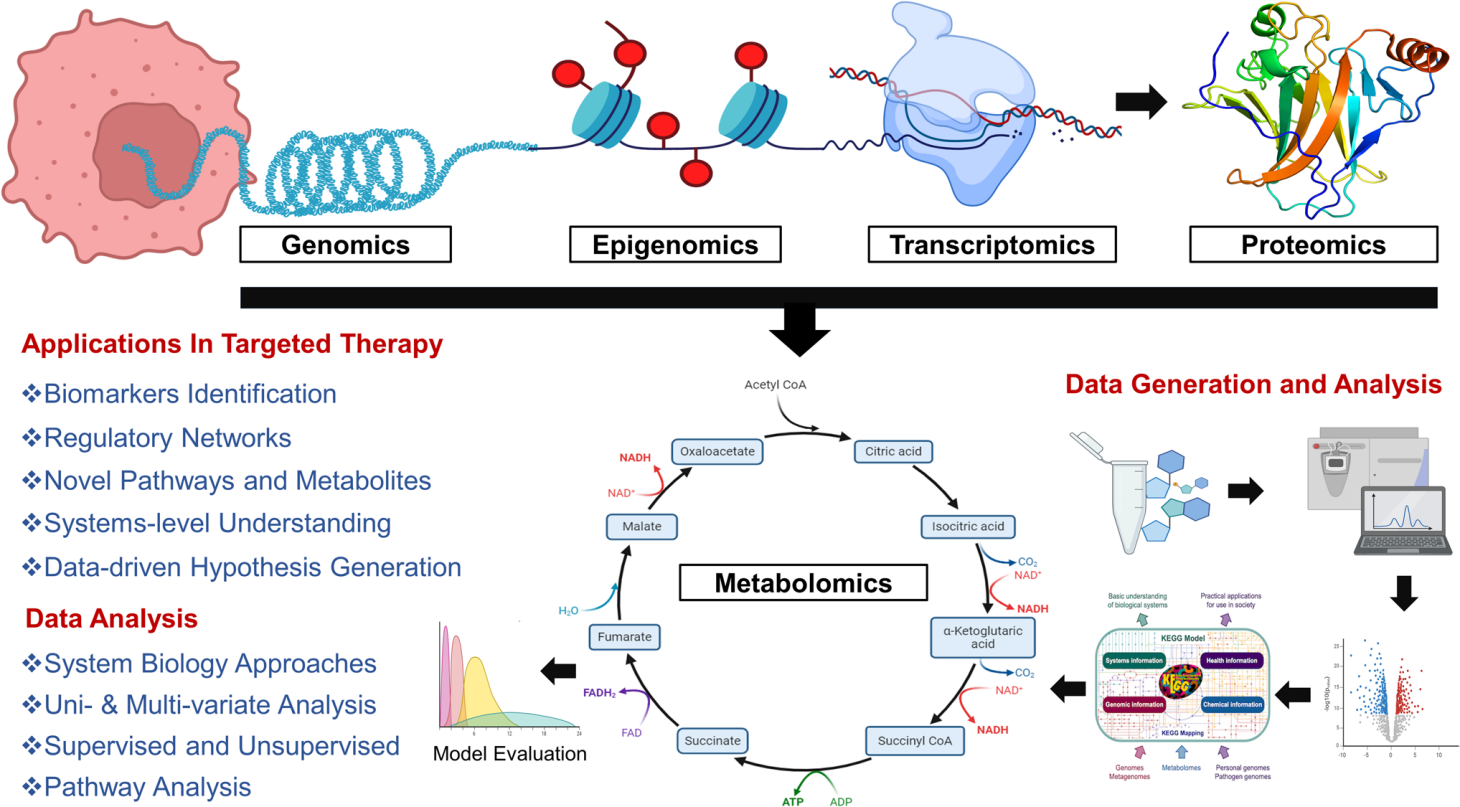
The workflow for integration of metabolomics with other omics for a holistic understanding of disease progression

Despite recent progress in omics technologies, the underlying genetic factors contributing to numerous metabolic phenotypes remain elusive. Metabolite biomarkers can be integrated with genomics and clinical parameters to enhance diagnostic accuracy or refine disease risk prediction models. Metabolites can also serve as intermediate phenotypes for genetic investigations, offering insights into underlying genetic mechanisms [162]. The integration of metabolomics data with either whole-exome sequencing or WGS-data presents a promising systematic strategy for pinpointing disease-causing variants and holds potential utility within the framework of a specific pathway under investigation [163]. Furthermore, at a more intricate biological and analytical level, metabolomics can be combined with various omic platforms, facilitating a comprehensive understanding of complex biological systems and interactions (Fig. 4).

The alterations in metabolite levels, perturbations in metabolic pathways, and the onset of disease states can be elucidated by assessing the epigenetic alterations. This approach offers molecular insights into the intricate interplay among genetic, epigenetic, and metabolic factors during the disease progression. Through the integration of epigenomic and metabolomic data, the intricate relationships between epigenetic alterations and metabolic pathways in disease pathogenesis can be uncovered. In recent years, metabolomics and epigenomics have experienced notable advancement as prominent molecular and analytical methodologies for biomarker identification [164, 165]. In the context of cancer, it is characterized by distinctive features such as metabolic reprogramming and epigenetic modifications, which play pivotal roles in tumor progression and are intricately interconnected with the tumor microenvironment and other molecular pathways [166]. Epigenetic modifications can directly influence the expression of metabolic genes, thereby altering cellular metabolism and contributing to disease phenotypes [167]. Conversely, metabolic alterations can impact epigenetic regulation by modulating the availability of metabolites involved in epigenetic modifications [166, 168]. The cross-talk between epigenetics and metabolism represents a dynamic interplay that shapes cellular physiology and disease susceptibility [169, 170]. DNA methylation stands out as a widely studied epigenetic mechanism with implications for cancer-related gene regulation. Methylation processes, occurring directly in promoter regions of cancer-related genes or histone residues, significantly influence DNA accessibility and gene expression regulation. The availability of methyl groups, primarily mediated by metabolites within the methionine and folate cycles, closely relates to DNA methylation processes [171]. Alterations in metabolic concentrations of the Tricarboxylic Acid (TCA) cycle intermediates, such as α-ketoglutarate (α-KG), succinate, fumarate, and acetyl-CoA, significantly impact chromatin-modifying enzymes, including 10–11 translocation enzymes and histone demethylases. These enzymes play crucial roles in catalyzing hydroxylation and demethylation processes, ultimately shaping the epigenetic landscapes of cancer. Additionally, metabolites such as succinate, fumarate, and α-KG, termed oncometabolites, accumulate in cancer cells due to mutations in fumarate hydratase and succinate dehydrogenase genes, further influencing epigenetic alterations [170, 172]. Overall, the regulation of tumor gene expression reflects a complex interplay between epigenetic enzymes and metabolic substrates, demonstrating the intricate mechanisms underlying cancer pathogenesis.

The conventional linear model of data progression from genes to transcripts, proteins, and metabolites is being reconsidered to recognize the intricate association of these network layers. Relying solely on single-level data often proves inadequate for fully understanding biological processes. For instance, fluctuations in metabolite concentrations may arise from downstream production or reduced metabolism from upstream reactions, making causal assessments challenging. Similarly, while transcripts provide insight into gene transcription, they do not convey the functionality or activity of resulting proteins [173]. Consequently, integrated Omics methodologies with transcriptomics and metabolomics emerging as commonly preferred combinations in contemporary investigations are being adopted to gain a more comprehensive understanding of disease progression with novel regulatory pathways and biomarkers [174]. This approach yields comprehensive datasets elucidating the interconnected metabolic and transcriptomic alterations. This integration will facilitate the identification of relationships between proteins and metabolites, thereby revealing molecular mechanisms derived from high-throughput data [175]. The metabolome contributes phenotypic measurements, serving as an anchor for the comprehensive global measurements obtained from the transcriptome, enhancing the overall analytical capacity of this integrated approach [174].

However, the integration of proteomics data are essential for bridging the gap between mRNA expression and metabolite abundance. Proteomics offers valuable insights into the pathophysiological mechanisms underlying disease conditions. In the context of cancer, alterations in metabolism may arise not solely from variations in protein levels but also from the modulation of enzyme activity [176]. The impacts of the latter remain imperceptible at the proteome level. The fluctuations in associated metabolites necessitate investigations through metabolomics. Nevertheless, the assessment of critical metabolites can effectively augment proteomics data, providing a more comprehensive understanding of the intricate metabolic processes at play. The reductionist methodologies treated intracellular signaling cascades as linear constructs, portraying involved molecules within discrete signal transduction pathways [177]. However, it is now recognized that diverse pathways engage in cross-talk, forming intricate networks that encompass both proteins and metabolites. Databases like the Kyoto Encyclopedia of Genes and Genomes (KEGG), The Human Metabolome Database (HMDB), and Reactome serve to map the regulation of enzymes and metabolites across various metabolic networks [178–180]. Consequently, the integration of proteomics and metabolomics offers a complementary data read-out, enhancing confidence in the interpretation of intricate molecular interactions.

## Foreseen Prospects and Obstacles

The future of omics data analysis holds tremendous promise but is accompanied by significant challenges. High-throughput omic techniques for biological sample analysis have become prevalent, and as a consequence, data sized at terabytes to petabytes are routinely generated by each analysis [181, 182]. The integration of this multi-dimensional omics data into a relevant biological context is challenging due to the volumes of the data and the variances in nomenclature across various data types [182]. The availability of extensive omics information has transformed the field of biology and sparked the development of a systems approach, which aims to enhance our comprehension of complex biological processes [119]. Various systems approaches enable the representation of molecular interactions and pathways, facilitating the elucidation of underlying biological mechanisms. However, challenges persist in interpreting and integrating multi-dimensional omics data, particularly in capturing temporal and spatial dynamics within biological systems.

Data standardization is pivotal in ensuring the quality and comparability of omics datasets across studies and platforms. Standardization protocols encompass various aspects, including data preprocessing, normalization techniques, and metadata annotation, aimed at harmonizing data structures and minimizing technical artifacts [183]. However, omics data exhibit inherent heterogeneity stemming from differences in experimental protocols, sample types, and analytical platforms, posing challenges in data integration and analysis [52]. Addressing data heterogeneity requires robust statistical methods and computational algorithms capable of accommodating diverse data types and mitigating batch effects [53, 94]. New technologies continue to emerge, offering novel avenues for omics data analysis, such as single-cell omics and spatial omics techniques, which promise more profound insights into cellular heterogeneity and spatial organization [184]. However, despite its transformative potential, omics data analysis is not exempted from inherent limitations and challenges. Ethical considerations loom large in omics data analysis, particularly concerning data privacy, ownership, consent, and fair data use. These factors hinder the exchange of accessible data and restrict opportunities for collaborative integration projects, which necessitate rigorous ethical assessments and transparent communication with research participants and stakeholders [185, 186].

Despite the transformative potential of omics data, several limitations and challenges persist. Technical limitations, such as data noise, missing values, and limited sample sizes, may compromise the reliability and generalizability of omics-derived insights [81, 187]. Moreover, the complexity of biological systems presents formidable challenges in deciphering causal relationships and predicting system behavior accurately [120]. Additionally, the scalability and interpretability of ML algorithms pose challenges in handling the vast volume and dimensionality of omics data and extracting meaningful biological signals effectively [11].

In navigating these complexities, several questions emerge. What strategies can be implemented to develop standardized pipelines for preprocessing, quality control, normalization, and integration of omics data from different platforms and technologies to ensure consistency and reproducibility? What techniques can be employed to quantify uncertainty in integrated omics analysis results and propagate errors throughout the analysis pipeline to provide more reliable estimates of biological significance? What strategies can be implemented to refine and expand dynamic network models for capturing the intricate interactions within biological systems, considering the temporal and spatial dimensions? Furthermore, how do we address the ethical dilemmas surrounding data privacy, consent, and data ownership in omics research? Addressing these questions necessitates interdisciplinary collaboration, methodological innovation, and ongoing dialogue among researchers, policymakers, and the broader scientific community. By confronting these challenges, omics data analysis holds the potential to revolutionize our understanding of complex biological and disease phenomena and drive innovation in targeted therapeutics and personalized medicine.

## Conclusion

The development of novel therapeutics entails significant expenses and is time-consuming. Computational approaches are helpful since they were critical to the success of many novel therapeutics, which has been a watershed in the research. Several limitations arise in the conventional algorithms due to the non-repeatability and difficulty in interpreting results, which can be exacerbated with the production of extensive, high-dimensional data, as the results are often sensitive to the specific methods and parameters used. This can lead to inconsistencies and discrepancies between studies, which can affect the overall reliability of the study. Additionally, advancements in ML algorithms continue to improve the robustness and interpretability of results. The systems biology approach incorporates the information retrieved from omics data with robust computational tools and algorithms to construct and portray biological network models that aid in establishing an illusionary biological system with characteristics that may be tweaked or rendered static and dynamic for novel biomarkers discovery. The advances in the development of robust computational algorithms and techniques have successfully generated predictions through computational models, further combined with experimental validation to bog down the time and cost of the drug discovery process.
